# Controlled Synthesis of Carbon Nanoparticles in a Supercritical Carbon Disulfide System

**DOI:** 10.3390/ma7010097

**Published:** 2013-12-27

**Authors:** Zhengsong Lou, Hongying Huang, Min Li, Tongming Shang, Changle Chen

**Affiliations:** 1 Laboratory of Precious Metal Processing Technology and Application, School of Applied Chemistry, Jiangsu University of Technology, Changzhou 213001, China; E-Mails: hhy@jstu.edu.cn (H.H.); shangtm@jstu.edu.cn (T.S.); 2 Chinese Academy of Science (CAS) Key Laboratory of Soft Matter Chemistry, Department of Polymer Science and Engineering, University of Science and Technology of China, Hefei 230026, China; E-Mail: liminkd@mail.ustc.edu.cn

**Keywords:** carbon nanoparticles, chemical synthesis, Raman spectroscopy

## Abstract

Carbon nanoparticles with large surface areas were produced by the reduction of carbon disulfide with metallic lithium at 500 °C. The carbon nanoparticles account for about 80% of the carbon product. The carbon nanoparticles were characterized by X-ray powder diffraction, field emission scanning electron microscopy, transmission electron microscopy, high resolution transmission electron microscopy and N_2_ physisorption. The results showed that carbon nanoparticles predominate in the product. The influence of experimental conditions was investigated, which indicated that temperature plays a crucial role in the formation of carbon nanoparticles. The possible formation mechanism of the carbon nanoparticles was discussed. This method provides a simple and efficient route to the synthesis of carbon nanoparticles.

## 1. Introduction

Nanostructured carbon materials have attracted tremendous attention due to their unique structures and superior properties. Carbon nanoparticles (CNPs) are of great interest for both fundamental studies and practical applications. CNPs have been widely used in supercapacitors [1], high-performance electrode materials in batteries [2] and excellent photoluminescent materials [3]. Specifically, CNPs with large surface areas can be readily functionalized, allowing efficient binding of biomolecules. Compared with traditional quantum dots and organic dyes, photoluminescent carbon nanomaterials have advantages due to their chemical inertness and lower toxicity [4]. Intense efforts have been invested in the production of CNPs. Bottom-up approaches such as pyrolysis [5–9], microwave plasma enhanced chemical vapor deposition [10,11], electrolysis in molten salt [12], graphitization of particles obtained by microemulsion polymerization [13], laser vaporization of a carbon pellet [14] and treatment in supercritical water [3,15] are the predominant process. It should be noted that these methods only generate raw products, which require either further purification (e.g., laser ablation or arc discharge) or the removal of catalysts (e.g., chemical vapor deposition).

We have synthesized diamond particles with size up to 460 μm by reduction of CO_2_ with metallic sodium or potassium at 440 °C [16,17]. Due to similar chemical and physical properties (polarity, supercritical phase, *etc*.) of carbon disulfide with carbon dioxide, we explored the possibilities of preparing diamond in carbon disulfide-alkali metals system. However, only graphite with no diamond was detected in the product. Moreover, large amount of CNPs were observed when the reaction was carried out at 500 °C. The reaction can be formulated as follows:

$${\text{CS}}_{2(\text{g})}+\;4{\text{Li}}_{\left(\text{l}\right)}\to \;{\text{C}}_{\left(\text{nanorods}\;+\text{graphite}\right)}+\;2{\text{Li}}_{2}{\text{S}}_{\left(\text{s}\right)}$$ (1)

We call this route the carbon bisulfide thermal reduction process, in which carbon bisulfide is used as the carbon source and Li is used as the reductant. After the reaction, Li_2_S reacted with hydrochloric acid to form LiCl which is soluble in water and thus it can easily be removed to get pure carbon material as the product. Based on emission scanning electron microscope (FESEM) and Transmission electron microscopic (TEM) anaylsis, CNPs account for about 80% of the carbon product, and the rest are graphite and amorphous carbon. The strategy is simple, efficient and affords products with high purity in high yield.

## 2. Results and Discussion

In a typical reaction, a certain amount of carbon disulfide and metallic lithium were placed in an autoclave, which was heated to 500 °C, and kept at this temperature for 8 h. After cooling the sample to room temperature, the solid product was collected and treated in 6.0 mol/L HCl aqueous solution. The precipitate was washed several times with distilled water, collected by centrifugation and dried at 80 °C overnight in a drying oven to yield the final product.

**Figure 1.**
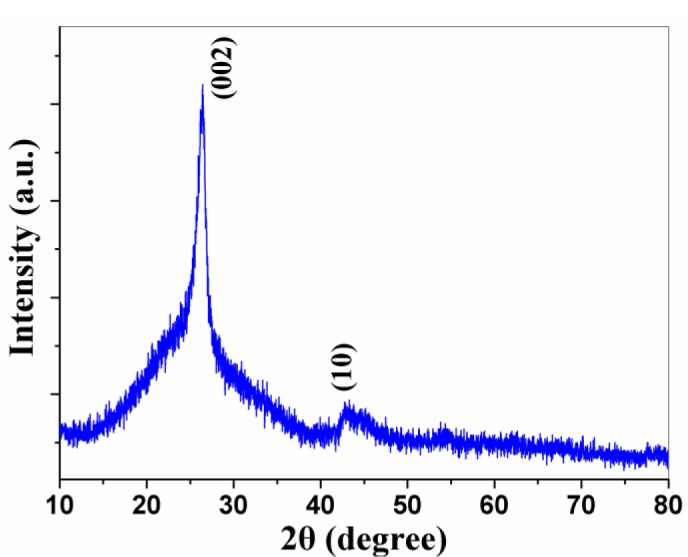
XRD pattern of the products after treatment with HCl aqueous solutions.

The crystallinity and structure of the black products was characterized by the powder XRD technique. Figure 1 shows the XRD pattern of the product after treated with 6.0 mol/L HCl aqueous solution. Reflections in the figure can be indexed to (002) and (10) of graphite (JCPDS Card Files, No. 41-1487). The (002) peak at 26.1° is shifted slightly from 2θ = 26.4° for graphite, corresponding to an increase in the spacing between the sp^2^ carbon layers from 0.337 nm for graphite to 0.341 nm for CNPs [18]. Also, the broad (10) diffraction originates from a two-dimensional lattice.

**Figure 2.**
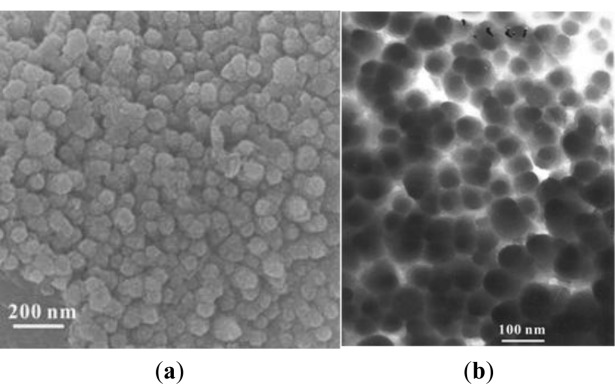

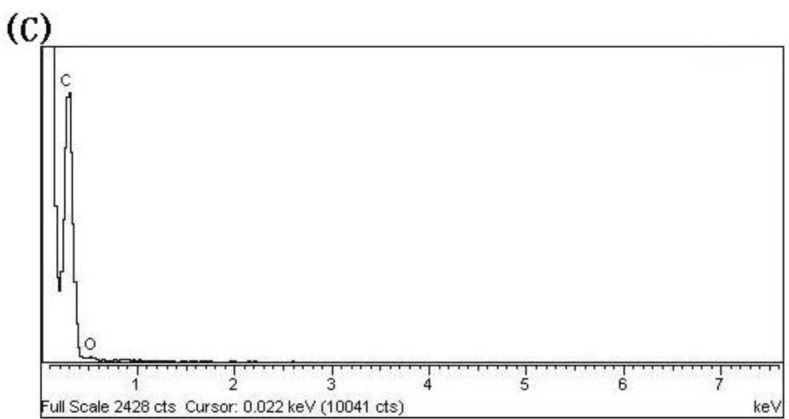
(**a**) FESEM image of the as-prepared product; (**b**) TEM micrographs of the carbon nanoparticles; (**c**) EDX of of the carbon nanoparticles.

Figure 2a shows a FESEM image of the sample, indicating the large numbers of CNPs obtained via this approach. The size of the CNPs is not uniform and ranges from several tens of nanometers to 150 nm. CNPS are not regular spheres and their surface is rough. We carried out SEM–EDX analysis of the CNPs shown in Figure 2c, and no sulfur was detected in it. As to elements other than carbon, the oxygen atoms may be due to the presence of O_2_ in the air. This suggested the high purity of the final product. Figure 2b is a typical TEM photograph of the CNPs. The particles in the sample appear to be irregular, with diameters ranging from 50 to 150 nm, which agrees very well with the FESEM result.

**Figure 3.**
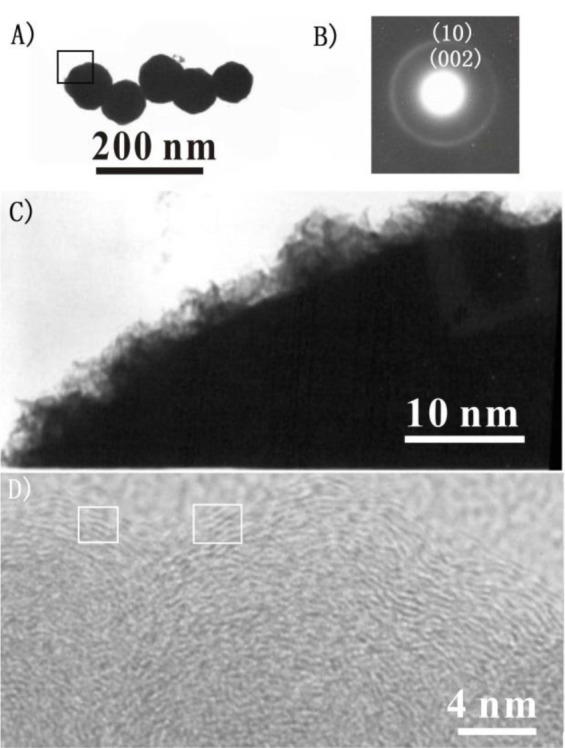
HRTEM pictures of the product. (**A**) TEM micrograph of the typical carbon nanoparticles; (**B**) Selected area electron-diffraction pattern of the carbon nanoparticles; (**C**) is the enlarged image of the rectangle area shown in (**A**). The magnified high-resolution image of the carbon nanoparticles; (**D**) HRTEM image of the product.

A more detailed investigation of as-prepared products was carried out by HRTEM. Figure 3A is a typical TEM image of the CNPs, and the selected area electron diffraction pattern (Figure 3B) of the CNPs comprises two diffraction rings corresponding to (002) and (10) reflections of graphite, confirming the graphitic shell structure of the CNPs, which is in agreement with the XRD result.

Figure 3C is the enlargement of the rectangle area of the nanoparticle shown in Figure 3A, in which fibre-like nanoprotrusions can be clearly observed, indicating the large surface area of the particles. The unique structure of this material may find applications in catalyst support and drug-delivery. For example, the CNPs is expected to have improved properties as adsorbent materials, because foreign substances might become more easily incorporated into the CNPs, with the fibrelike nanoprotrusions acting as active adsorption sites.

HRTEM analysis (Figure 3D) reveals that the CNPs consist of amorphous cores and crystalline shells. It was observed that the crystalline shell (the boxed area) has curved and nearly parallel lines, which are graphitic atomic planes separated by 0.34 nm consistent with the (002) plane lattice parameter of graphited carbon.

**Figure 4.**
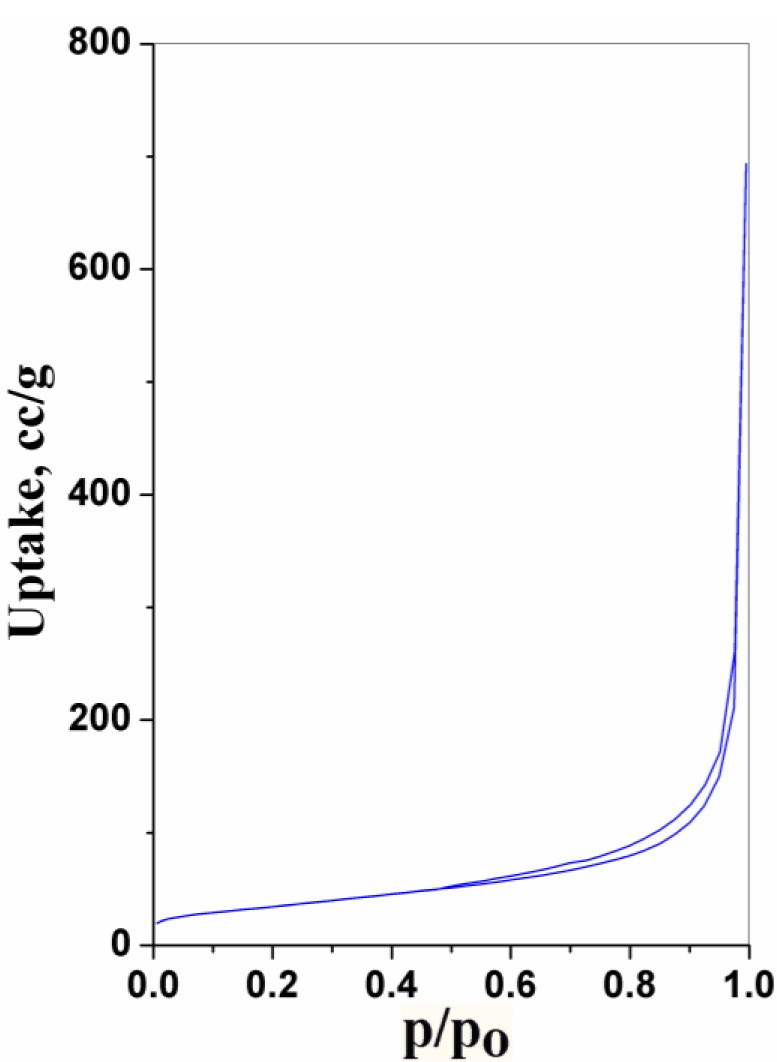
Nitrogen physisorption results of the carbon nanoparticles: adsorption/desorption isotherms.

The nitrogen adsorption isotherms of the CNPs obtained at 77 K are shown in Figure 4. The isotherms of the sample appears typically in Type IV characteristic, according to the IUPAC (The International Union of Pure and Applied Chemistry) classification, with hysteresis loops initiating from the medium relative pressures (*P*/*P*_0_ ~ 0.40), and closing near *P*/*P*_0_ ~ 1 [19]. BJH cumulative desorption surface area is 86.42 m^2^·g^−1^. The surface area is relatively high, which is in agreement with the HRTEM results.

**Figure 5.**
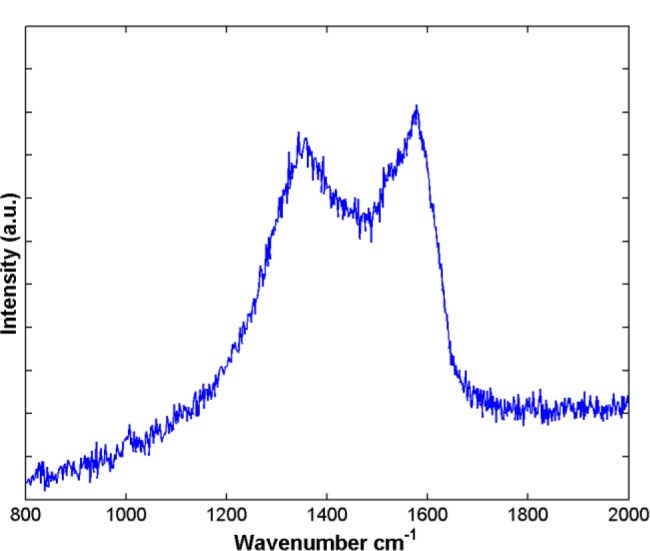
Raman spectrum of the products after treatment by HCl aqueous solutions.

Figure 5 shows the Raman spectrum of the sample. The spectrum presents two peaks centered at 1577 and 1356 cm^−1^, which are the typical characteristics of the graphitic carbon nanostructures. The peak at 1577 cm^−1^ (G band) corresponds to an E_2g_ mode of graphite and is related to the vibration of sp^2^-bonded carbon atoms in a 2-dimensional hexagonal lattice, such as in a graphite layer [20]. The peak at 1356 cm^−1^ (D band) is associated with vibrations of carbon atoms with dangling bonds in plane terminations of disordered graphite. The peak is quite high indicating that in the basal plane there exists two-dimensional disorder. Also, the presence of defects in the product is highly possible based on Raman analysis. In general, the relative intensity (*I*_D_/*I*_G_) ratio is proportional to the number of defects in graphitic carbon. The high *I*_D_/*I*_G_ ratio indicates that some defects are present in the CNPs. It is noted that a wide band instead of sharp peaks and an overlapped D and G band in the region of 1000–1700 cm^−1^, although the Raman selection rule is relaxed in amorphous structures, which suggests the carbon products as-synthesized are amorphous. However, it is not in agreement with the HRTEM images. The novel structure of the CNPs is likely to be responsible for this difference, which is also thought to arise from the presence of sp^3^ bonds that bind together with the sp^2^ bounded graphitic planes.

To investigate the possible catalytic role of the constituents from the stainless steel reactor, a reaction was carried out in a copper cell under the same experimental conditions. The same products were obtained. This rules out any possible catalytic role of any metal atoms from the stainless steel reactor.

To investigate the effect of reaction conditions on the formation of CNPs, a series of control experiments were carried out. It is found that reaction temperature played a critical role in the formation of these CNPs. No reaction was observed at temperatures lower than 280 °C and graphite and amorphous carbon were generated at 400 °C. At 500 °C, a large amount of the CNPs were formed, as shown in Figures 2 and 3. When the reaction is carried out at 600 °C, the main products were carbon nanorods and graphite [19]. Also in a reaction at 500 °C for 8 h without the addition of lithium, it was found that only amorphous carbon was formed; suggesting CNPs formation is related to the reduction of CS_2_ by metallic Li.

The metallic lithium melts at 180.5 °C and its boiling temperature is 1342 °C. The density of metallic lithium is 0.534 g/cm^3^, but the density of carbon disulfide is 1.263 g/cm^3^, so the molten metallic lithium floats on the surface of carbon disulfide. The critical point of carbon disulfide is characterized by the pressure and temperature *P*_c_ = 75 atm and *T*_c_ = 546 K, respectively. The pressure of reaction system at 500 °C is about 700 atm, which indicates CS_2_ is in a supercritical state, so the gas and liquid phases merge into a single supercritical phase, which serves as a novel medium for chemical reactions. Based on our experimental conditions and results, we describe a possible growth mechanism of the CNPs. In the supercritical CS_2_ system, liquid Li might form a nanoscale rough surface with nanoprotrusions (nanoprotrusions nanodroplets) at the growth temperature. The nanodroplets take the roles both as reductant for CS_2_ reduction and as templates for the growth of CNPs. It is suggested that molten lithium floating on the surface of carbon disulfide, form many Li nanodroplets, and CS_2_ molecules are reduced on Li droplets, resulting in the formation of CNPs. Detailed mechanism for the reduction of CS_2_ to carbon and the generation of CNPs is still progress.

## 3. Experimental Section

Metallic lithium and carbon disulfide were used as reactants to synthesize CNPs. The reaction was carried out in a stainless steel autoclave (14 mL), which is a 110-mm-long cylindrical tube with outer diameter of 85 mm and an inner diameter of 13 mm. In a typical reaction, 11 mL carbon disulfide and 1.0 g metallic lithium were placed in the cell at room temperature. The sealed autoclave was ultimately placed in a furnace, heated at a ramp rate of 5 °C per minute up to a reaction temperature at 500 °C, and kept at this temperature for 8 h. The reaction took place at an autogenic pressure depending on the amount of carbon disulfide added. After the autoclave cooled to room temperature in the furnace naturally, the dark solid product was collected and transferred to a beaker. The obtained sample was washed with absolute ethanol, to remove residual impurities, such as remaining CS_2_. Then, the product was treated in 6.0 mol/L HCl aqueous solutions and stirred in the acid solution at room temperature for 12 h to remove the Li_2_S formed. The mixture was then filtered, washed with deionized water several times until the filtrate turned out to be of neutral pH. Finally, the isolated solid carbon product was dried at 80 °C overnight in a drying oven. The solid precipitate, weighing 0.31 g (14% yield based on CS_2_) was confirmed to contain CNPs, graphite and amorphous carbon.

*Caution*: Metallic lithium releases flammable gases which may ignite spontaneously in contact with water and it causes severe skin burns and eye damage. Carbon disulfide is a highly flammable liquid that causes skin and severe eyes irritation, damage to organs through prolonged or repeated exposure and it is suspected of damaging fertility or the unborn child. Therefore, cautions should be paid during the whole operation process.

The X-ray diffraction (XRD) analysis was performed using a Rigaku (Rigaku Corporation, Akishima, Japan) D/max-γA X-ray diffractometer equipped with graphite monochromatized Cu Kα radiation (λ *=* 1.54178 Å). The morphology of the samples and energy dispersive X-ray analysis (EDX) were taken on a field FESEM (JEOL-6300F, JEOL Corporation, Akishima-shi, Japan, 15 kV). TEM images and electron diffraction (ED) patterns were taken on a Hitachi H-800 transmission electron microscope (Hitachi Corporation, Tokyo, Japan). The microstructure of CNPs was analyzed by high-resolution transmission electron microscopy (HRTEM, JEOL Corporation, Akishima-shi, Japan), which was performed on a JEOL-2010 transmission electron microscope using an accelerating voltage of 200 kV. N_2_ adsorption-desorption studies were carried out at 77 K with a static volumetric instrument Autosorb-1 (Quantachrome, Boynton Beach, FL, USA) to examine the porous properties of the sample. Samples were pretreated by outgassing in vacuum at 200 °C for at least 5 h. and the pore size distribution was evaluated from the desorption isotherms using the BJH method [21]. The Raman spectroscopy analysis was carried out on a LABRAM-HR Confocal Laser Micro-Raman Spectrometer (Bruker Corporation, Billerica, MA, USA) with an argon-ion laser at an excitation wavelength of 514.5 nm at room temperature.

## 4. Conclusions

In summary, CNPs have been synthesized at high yield by reduction of carbon disulfide with metallic lithium at 500 °C. FESEM, TEM, HRTEM, and N_2_ isotherm experiments revealed the morphology, size and fine structures of the product. CNPs with fibre-like nanoprotrusions and relatively high surface areas might find potential applications as catalyst carriers, lubricants, hydrogen storage materials, drug delivery materials, *etc*. [22,23]. Because of the simplicity and high yield of this route, it may find wider application in other fields.
